# Improved Intrinsic Nonlinear Characteristics of Ta_2_O_5_/Al_2_O_3_-Based Resistive Random-Access Memory for High-Density Memory Applications

**DOI:** 10.3390/ma13184201

**Published:** 2020-09-21

**Authors:** Ji-Ho Ryu, Sungjun Kim

**Affiliations:** 1 Electronics Engineering, Chungbuk National University, Cheongju 28644, Korea; jhryu13@naver.com; 2 Division of Electronics and Electrical Engineering, Dongguk University, Seoul 04620, Korea

**Keywords:** memristor, RRAM, nonlinearity, read margin, conduction mechanism, F–N tunneling, XPS

## Abstract

The major hindrance for high-density application of two-terminal resistive random-access memory (RRAM) array design is unintentional sneak path leakage through adjacent cells. Herein, we propose a bilayer structure of Ta_2_O_5_/Al_2_O_3_-based bipolar type RRAM by evaluating the intrinsic nonlinear characteristics without integration with an additional transistor and selector device. We conducted X-ray photoelectron spectroscopy (XPS) analysis with different etching times to verify Ta_2_O_5_/Al_2_O_3_ layers deposited on the TiN bottom electrode. The optimized nonlinear properties with current suppression are obtained by varying Al_2_O_3_ thickness. The maximum nonlinearity (~71) is achieved in a Ta_2_O_5_/Al_2_O_3_ (3 nm) sample. Furthermore, we estimated the comparative read margin based on the I-V characteristics with different thicknesses of Al_2_O_3_ film for the crossbar array applications. We expect that this study about the effect of the Al_2_O_3_ tunnel barrier thickness on Ta_2_O_5_-based memristors could provide a guideline for developing a selector-less RRAM application.

## 1. Introduction

The interest in developing an alternative storage device to overcome the scaling-limit issue has been extensively growing during the past two decades [1,2,3]. Among the potential candidates for next-generation non-volatile memory, resistive switching random-access memory (RRAM) has received significant attention in recent years due to its noticeable resistive switching characteristics, such as fast switching speed [4,5], low power consumption [6], complementary metal-oxide-semiconductor (CMOS) compatibility [7], and multifunctional applicability, which is suitable for non-volatile memory [8,9,10], logic in memory [11,12,13], and neuromorphic device applications [14,15,16,17]. The RRAM is generally composed of a simple and compact metal-insulator-metal (MIM) structure, which has a major specialty in minimizing cell size, as 4F^2^ (F is feature size for lithography) [18,19]. Recently, several cases have been reported of fabricating a crossbar array for high integration using RRAM cells in various ways [20,21,22]. However, the crossbar array structure including two terminal RRAM cells has the issue about reading disturbance between adjacent cells because of the sneak current issue. [23,24]. Several approaches including one transistor-one resistor (1T-1R) [25], one diode-one resistor (1D-1R) [26], and one selector-one resistor (1S-1R) [27] have been introduced to overcome this challenging issue. However, in terms of spatial integration, the intrinsic nonlinear characteristic in RRAM is more desirable than the above candidates. Numerous studies have analyzed the effect of nonlinear switching with various material structures [28,29,30,31,32]. However, additional efficient methods are available to optimize and utilize the selector-less property in more detail.

In this work, we propose the built-in nonlinear characteristics in Pt/Ta_2_O_5_/Al_2_O_3_/TiN devices to realize the selector-less RRAM application. First, we confirm the comparative X-ray photoelectron spectroscopy (XPS) analysis by etching for a top-down layer with information from the investigated material. Depending on the Al_2_O_3_ thickness, we investigate the detailed nonlinear switching performances in both positive and negative bias to ensure high nonlinearity. The read margin from I–V characteristics of the devices with different Al_2_O_3_ thickness is calculated using the half-bias scheme in crossbar array application. We suggest the conceptual physical mechanism in the oxide layer to explain the specific effect of Al_2_O_3_ modulation.

## 2. Experimental Procedure

**Figure 1:**
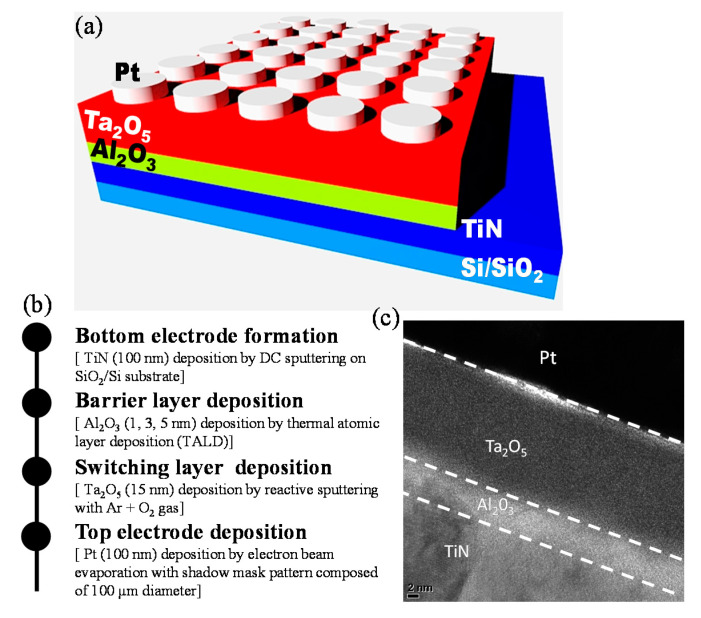
(**a**) Schematic illustration of Pt/Ta_2_O_5_/Al_2_O_3_/TiN device; (**b**) flow chart of device fabrication of the device; (**c**) Cross-sectional transmission electron microscopy (TEM) image of the device.

The schematic configuration of the Pt/Ta_2_O_5_/Al_2_O_3_/TiN structure on the SiO_2_/Si substrate is presented in Figure 1a, and the fabrication process is summarized in Figure 1b. First, we deposited the TiN as a bottom electrode (BE) on a SiO_2_/Si substrate by direct current (DC) sputtering with the thickness of approximately 100 nm. Afterward, the Al_2_O_3_ thin layer was deposited by thermal atomic layer deposition (TALD) at 350°C with different thicknesses for 1 nm, 3 nm, and 5 nm. We used trimethylaluminum (TMA) and ozone (O_3_) as a precursor for Al and O, respectively [33]. Then we deposited 15-nm-thick tantalum oxide (Ta_2_O_5_) as a main switching layer on the Al_2_O_3_ film by reactive DC sputtering from a tantalum target with Ar (8 sccm) and O_2_ (12 sccm) at room temperature. Finally, the 100-nm-thick Pt top electrode (TE) was deposited by e-beam evaporation and patterned through a shadow mask containing a circular pattern with a diameter of 100 µm. We observed the cross-sectional image from the transmission electron microscopy (TEM) in Figure 1c. The electrical properties in the DC sweep and transient modes were measured using a semiconductor parameter analyzer (Keithley 4200-SCS and 4225-PMU ultrafast module, Solon, OH, USA).Transmission electron microscope (TEM) and energy-dispersive X-ray spectroscopy (EDS) was conducted by the JEOL (JEM-2100F, Tokyo, JAPAN). Moreover, we applied a voltage to the Pt TE, while the TiN BE was grounded during the DC and pulse measurement.

## 3. Results and Discussion

**Figure 2:**
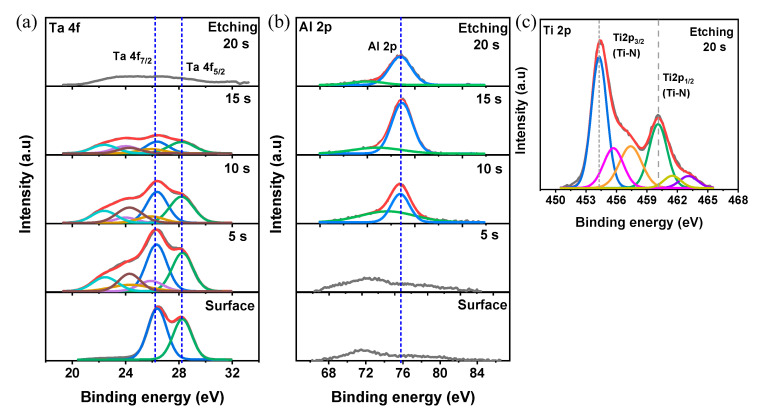
XPS analysis by Ar^+^ etching of Ta_2_O_5_/Al_2_O_3_/TiN layer: (**a**) Ta 4f spectra of the Ta_2_O_5_ film; (**b**) Al 2p spectra of the Al_2_O_3_ film; (**c**) Ti 2p spectra of the TiN bottom electrode.

In order to identify the chemical composition with a binding energy of Ta_2_O_5_/Al_2_O_3_/TiN structure, we carried out the XPS analysis to verify the comparative atomic spectra by Ar^+^ etching from the surface. The detailed XPS working conditions are summarized in Figure S1. Figure 1a–c shows the XPS spectra of Ta 4f, Al 2p, and Ti 2p, respectively. Ta 4f_7/2_ and Ta 4f_5/2_ peaks were centered at 26.3 eV and 28.2 eV, respectively, at the surface (0 s) [34]. Subsequently, the suboxide was detected after 5 s because of the Ta-O-Al bond near the Ta_2_O_5_/Al_2_O_3_ interface [35]. For the Al 2p peaks shown in Figure 2b, until the etching for 10 s, the Al 2p peak was undetectable because of the bulk Ta_2_O_5_ layer. The Al 2p peak was detected with 10 s after etching, indicating the Al^3+^ state (75.8 eV) of stoichiometric Al_2_O_3_ [36]. The second peak with low intensity is related to combination of Ta 4f and Al 2p. After 20 s, the sub-oxide peaks at a lower binding energy frequently appeared because of the formation of Al-O-Ti near the Al_2_O_3_/TiN interface. The Ti 2p spectra contains the dominant two doublets of Ti2p_3/2_ (454.2 eV) and Ti2p_1/2_ (460.15 eV) that stem from TiN in Figure 2c [37]. The other doublets at the low binding energy were affected by a reaction in the Al_2_O_3_/TiN interface, such as TiON.

**Figure 3:**
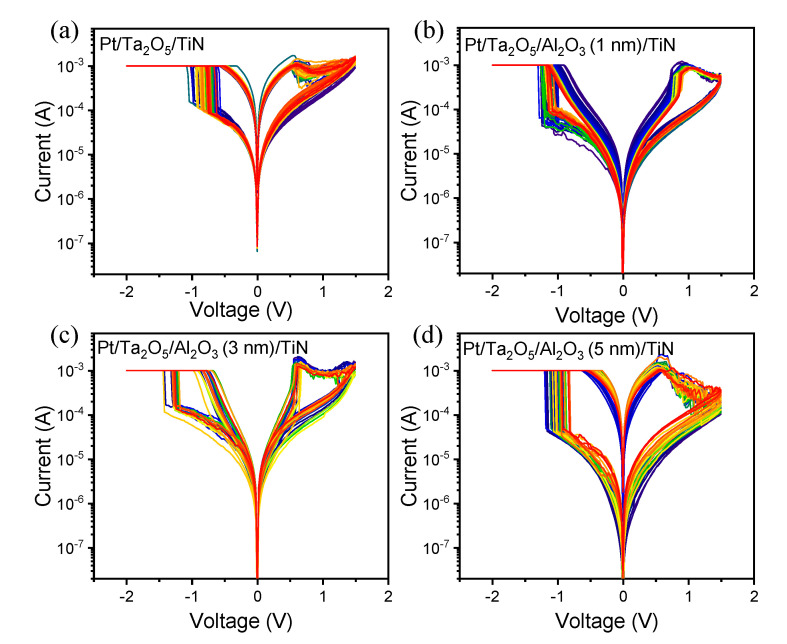
Experimental *I*–*V* results of (**a**) Pt/Ta_2_O_5_/TiN; (**b**) Pt/Ta_2_O_5_/Al_2_O_3_ (1 nm)/TiN; (**c**) Pt/Ta_2_O_5_/Al_2_O_3_ (3 nm)/TiN; (**d**) Pt/Ta_2_O_5_/Al_2_O_3_ (5 nm)/TiN devices.

To activate the oxygen ions/vacancies for the filament formation, a one-step electroforming process was induced to make the low-resistance state (LRS) while limiting the compliance current (CC) of 1 mA, as in Figure S2. Note that the electroforming voltage increased with the thickness of the Al_2_O_3_ layer. The CC was confined to 1 mA in order to prevent the device from permanent breakdown during the electroforming process. In Figure 3a–d, the experimental DC *I*–*V* characteristics of Pt/Ta_2_O_5_/TiN, Pt/Ta_2_O_5_/Al_2_O_3_ (1 nm)/TiN, Pt/Ta_2_O_5_/Al_2_O_3_ (3 nm)/TiN, and Pt/Ta_2_O_5_/Al_2_O_3_ (5 nm)/TiN devices are investigated to verify the nonlinear tendency depending on the thickness of Al_2_O_3_ layer. The results suggest that inserting an Al_2_O_3_ layer from 1 nm to 3 nm reinforces the nonlinear switching characteristics during the multiple cycles. In particular, the current increases abruptly, and then decrease gradually with the voltage sweep in a positive region, indicating that it is like a complementary resistive switching (CRS) curve. Note again that the current must be suppressed at low voltage to obtain a nonlinear curve. Therefore, it is important that the Al_2_O_3_ tunnel barrier should be maintained with the insulated property in the LRS (discussed in more detail later). The nonlinear property in the LRS loses for the Pt/Ta_2_O_5_/Al_2_O_3_ (5 nm)/TiN device because a high forming voltage makes the current overshoot [38,39]. It should be noticed that the optimized Al_2_O_3_ thickness between the Ta_2_O_5_ and TiN layer is the most important factor in ensuring the nonlinear characteristics after the electroforming process.

**Figure 4:**
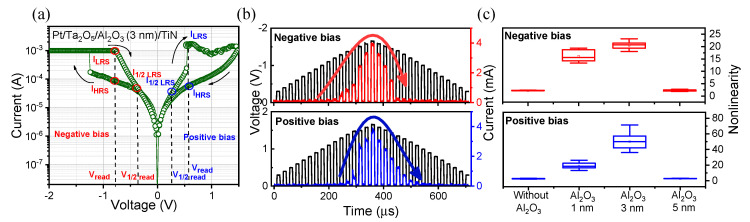
(**a**) Nonlinear *I–V* curve of Pt/Ta_2_O_5_/Al_2_O_3_ (3 nm)/TiN selector-less RRAM device. (**b**) Pulse responses of suppressed characteristics in Pt/Ta_2_O_5_/Al_2_O_3_ (3 nm)/TiN device. (**c**) Comparison box plot of nonlinear parameter in both polarities.

To specifically analyze the nonlinear characteristics of the Pt/Ta_2_O_5_/Al_2_O_3_ (3 nm)/TiN device, the SET process occurs with the CC of 1 mA by applying −2 V to turn into the LRS. On the other hand, the RESET process occurs by sweeping from 0 V to 1.5 V to turn back to high resistance state (HRS). The nonlinear *I*–*V* characteristics, which indicate the suppression of the current in a low-voltage region, are clearly demonstrated by the Pt/Ta_2_O_5_/Al_2_O_3_ (3 nm)/TiN device structure in Figure 4a. The nonlinearity is defined as the value of the current at read voltage (*V*_read_) divided by the current at half read voltage (*V*_1/2read_). In Figure 4b, the pulse current responses are observed to demonstrate the nonlinear property of the Pt/Ta_2_O_5_/Al_2_O_3_(3 nm)/TiN device. The currents are significantly suppressed when the applied voltages are low (<|1 V|). Figure 4c shows the statistical distribution of the nonlinearity in both polarities for 4 devices. Especially, the maximum nonlinearity (~71) is achieved by the Pt/Ta_2_O_5_/Al_2_O_3_(3 nm)/TiN device in a positive bias. The nonlinearity can be improved by inserting an Al_2_O_3_ layer with appropriate thickness in both polarities.

**Figure 5:**
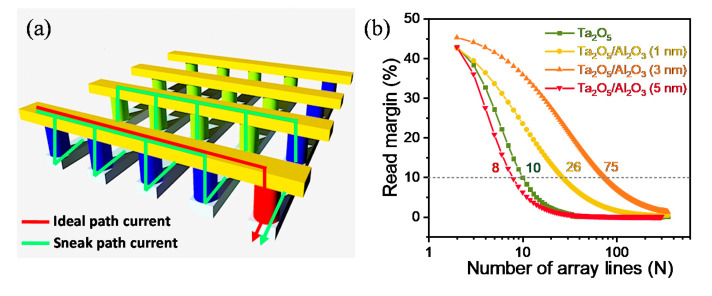
(**a**) Schematics of two-terminal RRAM based crossbar array configuration for high integrated application. (**b**) Calculated read margin as a function of number of array line for four devices.

The most effective use of the nonlinear characteristics in the Pt/Ta_2_O_5_/Al_2_O_3_/TiN device is the suppression of the sneak path to implement high-density crossbar arrays in Figure 5a. The RRAM cells with high nonlinearity have a strong immunity to overcome the sneak path interference [40]. The read margin is considered to evaluate the relationship between the array density and nonlinear behaviors in detail. Note that the read margin degrades with the increasing number of word lines because of a high sneak path current through the adjacent cells with increasing array size [41]. For the calculation based on Kirchhoff’s circuit law, the virtual two-terminal passive crossbar arrays (N rows × N columns) can be simplified as the equivalent circuit and calculated from the measured I–V characteristics with a half-bias read scheme [42]. The detailed process of calculation for the read margin is explained in Figure S3. Figure 5b presents the read margin by a unified half-bias scheme in Pt/Ta_2_O_5_/TiN device and Pt/Ta_2_O_5_/Al_2_O_3_(1, 3, 5 nm)/TiN devices. The array size (N rows × N columns) is extracted by 10% criteria to evaluate a reliable sensing margin. Compared to the Pt/Ta_2_O_5_/TiN device and Pt/Ta_2_O_5_/Al_2_O_3_(1, 5 nm)/TiN devices, the Pt/Ta_2_O_5_/Al_2_O_3_ (3 nm)/TiN device has a higher read margin (~71) because of the highest nonlinearity through optimization of the Al_2_O_3_ thickness.

**Figure 6:**
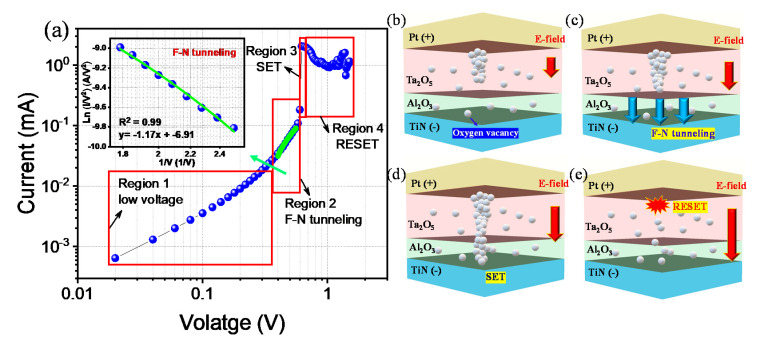
(**a**) Independent four regions of Pt/Ta_2_O_5_/Al_2_O_3_ (3 nm)/TiN device by differentiating the conduction process. (Inset shows the fitting result of Fowler–Nordheim (F–N) tunneling to evaluate the linearity in the fitting region). Conducting path evolution of Pt/Ta_2_O_5_/Al_2_O_3_ (3 nm)/TiN device in the positive bias. (**b**) high-resistance state (HRS); (**c**) FN tunneling in region 2; (**d**) LRS in region 3; (**e**) reset process in region 4.

To analyze the possible conducting mechanism process and the filamentary model of nonlinear characteristics in the Pt/Ta_2_O_5_/Al_2_O_3_/TiN device, we divided the experimental *I–V* curve in a positive bias into 4 regions to express the movement of oxygen vacancies in oxide layers (Figure 6a). The slope in log-log fitting and the current is at a low voltage in region 1 because the Al_2_O_3_ layer somewhat insulates the LRS. The Al_2_O_3_ tunnel barrier layer acts as the main role in nonlinear characteristics, by suppressing the current at low voltage. When the oxygen vacancies are increased by an increased electric field (E-field), Fowler–Nordheim (F–N) tunneling, which is one of the well-known conduction mechanisms in the thin-film dielectric [43], can be the dominant conduction mechanism in Figure 6c. It is also confirmed that the *I–V* curve in region 2 is well matched with ln (*I/V^2^*) vs. *1/V* fitting for F–N tunneling in Figure 6a [44]. When the electric field is strongly applied inside the Al_2_O_3_ tunnel barrier, the barrier viewed from the metal electrode is deformed into a triangle, so that the movement of the carrier becomes easier. In other words, the effective Al_2_O_3_ barrier becomes thinner. At the end of saturation with the more increased E-field, the Al_2_O_3_ also has become activated to form a conducting filament with high conductivity, and the device is changed to LRS for the set process in region 3, as shown in Figure 6d. Then finally, in Figure 6e, the overinflated E-field elicits the filament breakage in Figure 6e. The temporal LRS turns into HRS for a reset transition. These experimental results are well matched with previously reported research [45,46,47,48].

## 4. Conclusions

In summary, we proposed the intrinsic nonlinear characteristics in a Pt/Ta_2_O_5_/Al_2_O_3_/TiN RRAM device considering the Al_2_O_3_ thickness effect. The optimized bilayer device (3-nm-thick-Al_2_O_3_) shows better nonlinearity. The improved read margin in Al_2_O_3_ inserted devices are verified by the half-bias scheme simulation in cross-point structure. The XPS study verified the chemical state/element of the Ta_2_O_5_/Al_2_O_3_/TiN layer. The nonlinear properties are explained by the E-field-dependent conducting filament model process. This result can provide the feasibility of the selector-less RRAM approach in cross-point array structure.

## Supplementary Materials

The following are available online at https://www.mdpi.com/1996-1944/13/18/4201/s1, Figure S1: Summary of the XPS working condition, Figure S2: Comparison of electroforming voltages, Figure S3: The simplified circuits for read margin in crossbar array structure.
