# Supplementary material for: Improved Intrinsic Nonlinear Characteristics of Ta2O5/Al2O3-Based Resistive Random-Access Memory for High-Density Memory Applications
Source: Materials (Basel). 2020 Sep 21;13(18):4201. doi: 10.3390/ma13184201 (PMC7560462; doi:10.3390/ma13184201)
Supplement: Supplementary file 1 [file materials-13-04201-s001.pdf]

Supplementary Materials

Improved Intrinsic Nonlinear Characteristics of Ta_2O_5/Al_2O_3-Based Resistive Random-Access Memory for High-density Memory Applications

Ji-Ho Ryu [1] and Sungjun Kim [2,*]

1 Electronics Engineering, Chungbuk National University, Cheongju 28644, Korea; jhryu13@naver.com
2 Division of Electronics and Electrical Engineering, Dongguk University, Seoul 04620, Korea
* Correspondence: sungjun@dongguk.edu

Received: 13 August 2020; Accepted: 17 September 2020; Published: date

---

(a) **Working condition of X-ray Photoelectron Spectrometer (XPS) System by ThermoFisher Scientific**

- ❑ Energy resolution : ≤ 0.5 eV FWHM
- ❑ Sensitivity for narrow scan : 1 MCPS @ 0.6eV or better
- ❑ Minimum spot size : 30 μm or better
- ❑ Image resolution : 30 μm or better
- ❑ Monatomic and Gas cluster ion source (MAGCIS)
- ❑ Vacuum transfer Vessel / Sample bias module

Figure S1. (a) Summary of the XPS working condition.

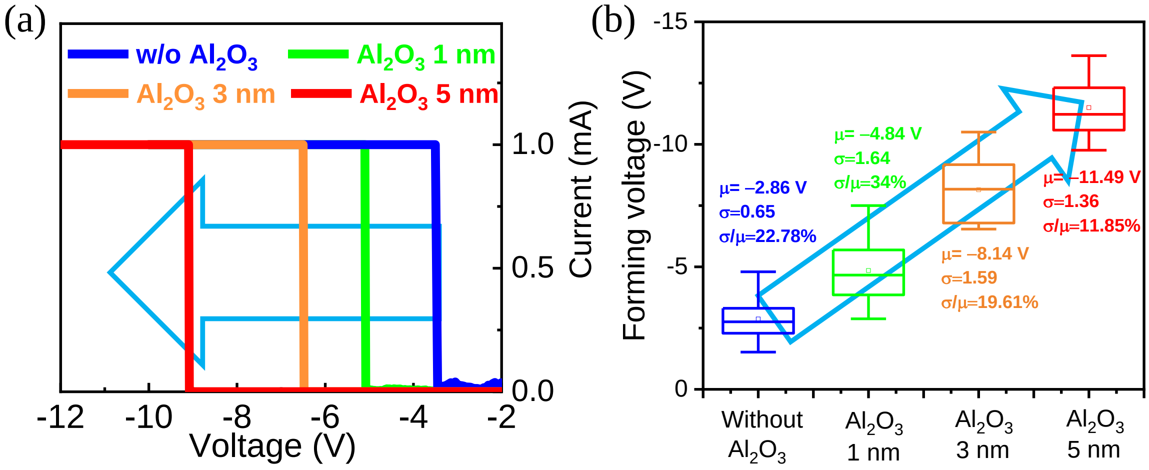

Figure S2. (**a**) Comparison of electroforming voltages (**b**) the distribution of electroforming voltages for four devices.

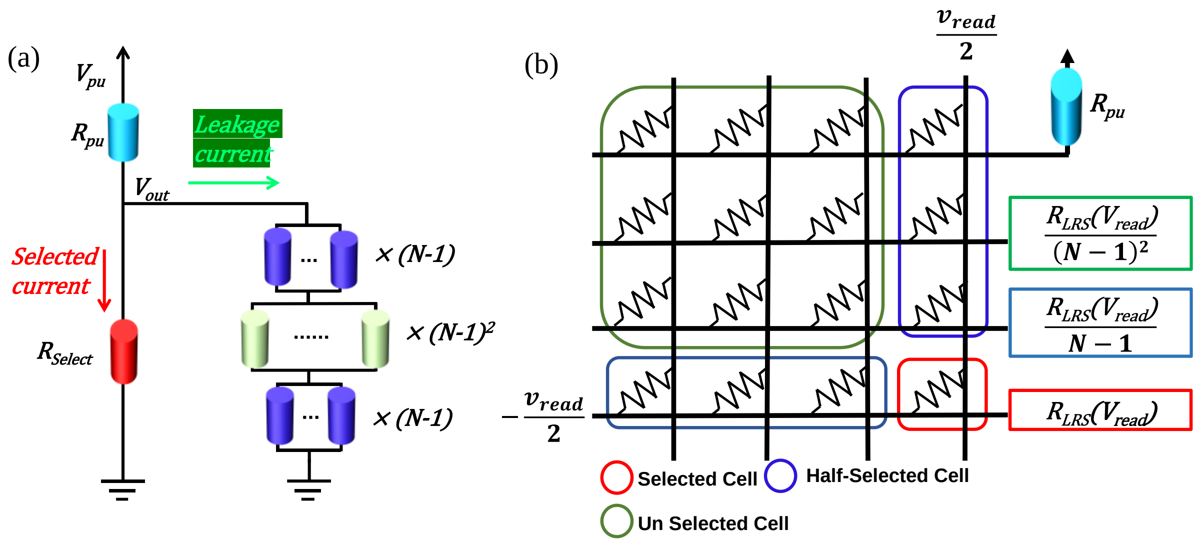

Figure S3. (a) Equivalent circuit of three regions and pull-up resistance in a cross array structure. (**b**) Half-bias read scheme with three different cell states.

The detailed calculation process for read margin was understood by simplified equivalent circuits in Figure S3a and determined by the half bias scheme in a N × N crossbar array as indicated in Figure S3b. Based on this electrical schematic diagram under the parallel calculation by the Kirchhoff equation, the total sneak resistance can be obtained as follows:

$$R_{sneak} = \frac{R_{LRS}^{sneak}}{N-1} + \frac{R_{LRS}^{sneak}}{(N-1)^2} + \frac{R_{LRS}^{sneak}}{N-1} \quad (1)$$

The sensing resistance in HRS and LRS are calculated from the followed equation:

$$R_{HRS,\ sensing} = \frac{R_{HRS}^{select} \times R_{sneak}}{R_{HRS}^{select} + R_{sneak}} \quad (2)$$

$$R_{LRS,\ sensing} = \frac{R_{LRS}^{select} \times R_{sneak}}{R_{LRS}^{select} + R_{sneak}} \quad (3)$$

The normalized read voltage margin (ΔV) is defined as follows:

$$\Delta V\ (\%) = \frac{V_{out,\ HRS}}{V_{pu}} - \frac{V_{out,\ LRS}}{V_{pu}} = \frac{R_{pu}}{R_{HRS,\ sensing} + R_{pu}} - \frac{R_{pu}}{R_{LRS,\ sensing} + R_{pu}} \times 100\ (\%) \quad (4)$$
